# Preparation of Antimicrobial Coatings from Cross-Linked Copolymers Containing Quaternary Dodecyl-Ammonium Compounds

**DOI:** 10.3390/ijms222413236

**Published:** 2021-12-08

**Authors:** Denisa Druvari, Aggeliki Antonopoulou, Georgia C. Lainioti, Alexios Vlamis-Gardikas, Georgios Bokias, Joannis K. Kallitsis

**Affiliations:** Department of Chemistry, University of Patras, 26504 Patras, Greece; druvari@upatras.gr (D.D.); agg.ant9@hotmail.com (A.A.); avlamis@upatras.gr (A.V.-G.); bokias@upatras.gr (G.B.); kallitsi@upatras.gr (J.K.K.)

**Keywords:** antimicrobial materials, polymeric coatings, cross-linking reaction, quaternary ammonium compounds, carboxyl groups, epoxy groups

## Abstract

One of the concerns today’s societies face is the development of resistant pathogenic microorganisms. The need to tackle this problem has driven the development of innovative antimicrobial materials capable of killing or inhibiting the growth of microorganisms. The present study investigates the dependence of the antimicrobial activity and solubility properties on the hydrophilicity/hydrophobicity ratio of antimicrobial coatings based on quaternary ammonium compounds. In this line, suitable hydrophilic and hydrophobic structural units were selected for synthesizing the antimicrobial copolymers poly(4-vinylbenzyl dimethyldodecylammonium chloride-co-acrylic acid), P(VBCDDA-co-AA20) and poly(dodecyltrimethylammonium 4-styrene sulfonate-co-glycidyl methacrylate), P(SSAmC_12_-co-GMA20), bearing an alkyl chain of 12 carbons either through covalent bonding or through electrostatic interaction. The cross-linking reaction of the carboxylic group of acrylic acid (AA) with the epoxide group of glycidyl methacrylate (GMA) of these two series of reactive antimicrobial copolymers was explored in blends, obtained through solution casting after curing at various temperatures. The release of the final products in pure water and NaCl 1 M solutions (as analyzed by gravimetry and total organic carbon, TOC/total nitrogen, TN analyses), could be controlled by the coating composition. The cross-linked polymeric membranes of composition 60/40 *w*/*w* % ratios led to 97.8 and 99.7% mortality for *Escherichia coli* (*E. coli*) and *Staphylococcus aureus* (*S. aureus*), respectively, whereas the coating 20/80 *w*/*w* % resulted in 96.6 and 99.8% cell reduction. Despite the decrease in hydrophobicity (from a 16- to a 12-carbon alkyl chain), the new materials maintained the killing efficacy, while at the same time resulting in increased release to the aqueous solution.

## 1. Introduction

Bacterial infection is a major problem for many areas, especially human health. The growing concern about diseases caused by pathogenic microorganisms has created a great need for innovative antimicrobial materials that can kill or inhibit the growth of pathogens. These materials serve valuable purposes in healthcare, personal care, food management, hygiene products, biomedical devices and surfaces that are highly prone to microbial contamination leading to disease [1,2]. Antimicrobial materials may be used as coatings on medical devices or surgical instruments, on materials that may contact the human body, such as implants or biomedical devices [3,4] and also as antifouling coatings for marine applications [5,6].

Polymeric materials with antimicrobial properties (polymeric biocides), are of interest for the academic and industrial communities [7]. The use of polymeric biocides may enhance the effectiveness of antimicrobials and may contribute to the minimization of environmental problems by reducing the residual toxicity of antimicrobials, while at the same time increasing their effectiveness and selectivity and extending their shelf life [1].

Antimicrobial cationic polymers have attracted attention in the field of antimicrobial polymers due to their high efficacy in the inhibition of the growth of drug-resistant microbes. An overview of their antimicrobial mechanism shows that these polymers are initially adsorbed primarily to the membrane of pathogenic microbes with the help of their cationic groups. In a second step, the hydrophobic groups of the cationic polymers disrupt the bacterial membranes leading to the death of the microbes. Therefore, both the cationic part and the hydrophobic groups of the cationic polymers are essential for their antimicrobial properties [3,8,9,10].

Many polymeric biocides are based on quaternary ammonium compounds (QACs) [11]. QACs are used for contact- or release-based applications since the quaternary groups can be either covalently attached to the polymer chain or electrostatically linked to the opposite ions of a polyanion. The use of environmentally acceptable biocidal materials such as those based on quaternary nitrogen compounds represents a research approach towards antimicrobial applications [12].

The antimicrobial efficiency of polymers bearing QACs is affected by the polymer charge density and the alkyl chain length of the cationic moieties [13,14]. Quaternary ammonium groups with ten or more carbon atoms can interact more strongly with the lipid bilayer of microbial membranes, thus leading to increased bactericidal activity compared to low alkyl groups [15,16,17]. However, the excessive chain length may have disadvantages: superhydrophobic structures may lead to strong aggregation between polymers, thus weakening their biocidal action while increased hydrophobicity may also lead to increased hemolytic activity.

The ratio between cationic and hydrophobic components in a polymer structure may also affect the biocidal activity of polymers bearing QACs. Excessive hydrophobicity causes high toxicity and poor solubility, while compounds with very low hydrophobicity are weak antibacterials and tend to cause erythrocyte aggregation (hemagglutination). Between these two extremes, there are certain compositions that provide the desired balance of hydrophobic and cationic character. The fundamental principle is to include the minimum amount of hydrophobic content required to impart antibacterial activity [18,19,20,21].

Dodecyltrimethylammonium bromide (DTAB) has been used as antimicrobial agent due to its better antimicrobial activity against seven different types of bacteria. According to the literature, using a one-step technology (i.e., antimicrobial agent DTAB in a spinning solution), a DTAB-modified antimicrobial hybrid membrane was obtained on the surface of the fibers. Antimicrobial activity has been demonstrated even at low DTAB concentrations (0.5% by weight) [22,23].

Another dodecyl compound used for polymer alkyl chain modification is *N*,*N*-dimethyldodecylamine (DDA). Studies investigating the effects of DDA on enzymes and growing *Escherichia coli* (*E. coli*) cells showed that DDA induces lysis in growing cells at rates associated with drug concentration, pH and previous growth rate. Plasma membrane damage by DDA resulted in the loss of low molecular weight metabolites and cofactors while the intracellular synthesis of wall components precursors stopped. DDA appears to mimic penicillin because its main site of action is the cellular membrane, inhibiting cell wall growth and causing the lysis of growing cells [24,25,26].

Our research group has been involved in the development of copolymers bearing immobilized and releasable quaternary ammonium groups with alkyl chains of 16-carbon atoms. In our previous works, the antimicrobial activity of such copolymers was investigated on cultures of the Gram-negative *Pseudomonas aeruginosa* (*P. aeruginosa*), and *E. coli* and the Gram-positive *Staphylococcus aureus* (*S. aureus*) and *Enterococcus faecalis* (*E. faecalis*) bacteria [27]. Moreover, we have also tested the antifouling efficiency of cross-linked membrane and films prepared from reactive antimicrobial copolymers bearing alkyl chains with 16-carbon atoms [28,29,30]. In the present work, our aim was to explore the possibility of using shorter alkyl chains of 12-carbon atoms for the preparation of effective cross-linked antimicrobial membranes/coatings and test the dependence of the antimicrobial activity and solubility properties of the polymers on the hydrophilicity/hydrophobicity ratio. Therefore, we used statistical copolymers with suitable hydrophilic and hydrophobic structural units, namely poly(4-vinylbenzyl chloride-co-acrylic acid), P(VBC-co-AA20), and poly(sodium 4-styrenesulfonate-co-glycidyl methacrylate), P(SSNa-co-GMA20). Quaternary ammonium units bearing an alkyl group of 12-carbons, were introduced in these copolymers either through covalent bonding (reaction of *N*,*N*-dimethyldodecylamine, DDA, with the vinylbenzyl chloride, VBC unit) or through electrostatic interactions (exchange of Na^+^ cation of sodium 4-styrenesulfonate, SSNa) unit with the dodecyltrimethylammonium cation, AmC_12_, of dodecyltrimethylammonium bromide, DTAB). The synthesized statistical copolymers, poly(4-vinylbenzyl dimethyldodecylammonium chloride-co-acrylic acid), P(VBCDDA-co-AA20) and poly(dodecyltrimethylammonium 4-styrene sulfonate-co-glycidyl methacrylate), P(SSAmC_12_-co-GMA20), were cross-linked through the reaction of the functional carboxylic (AA) and epoxide (GMA) groups and studied as membranes or coatings on glass surfaces. The biocidal efficacy of the 12-carbon compounds was compared to that of the respective 16-carbon compound of our previous published work [11]. In addition, the polymeric films and coatings prepared in the present work were studied in terms of their stability in aqueous environment and salt solutions to simulate the sea environment for potential use in relevant applications.

## 2. Results and Discussion

### 2.1. Synthesis and Characterization of P(VBCDDA-co-AA20) and P(SSAmC_12_-co-GMA20) Antimicrobial Copolymers

**Figure 1:**
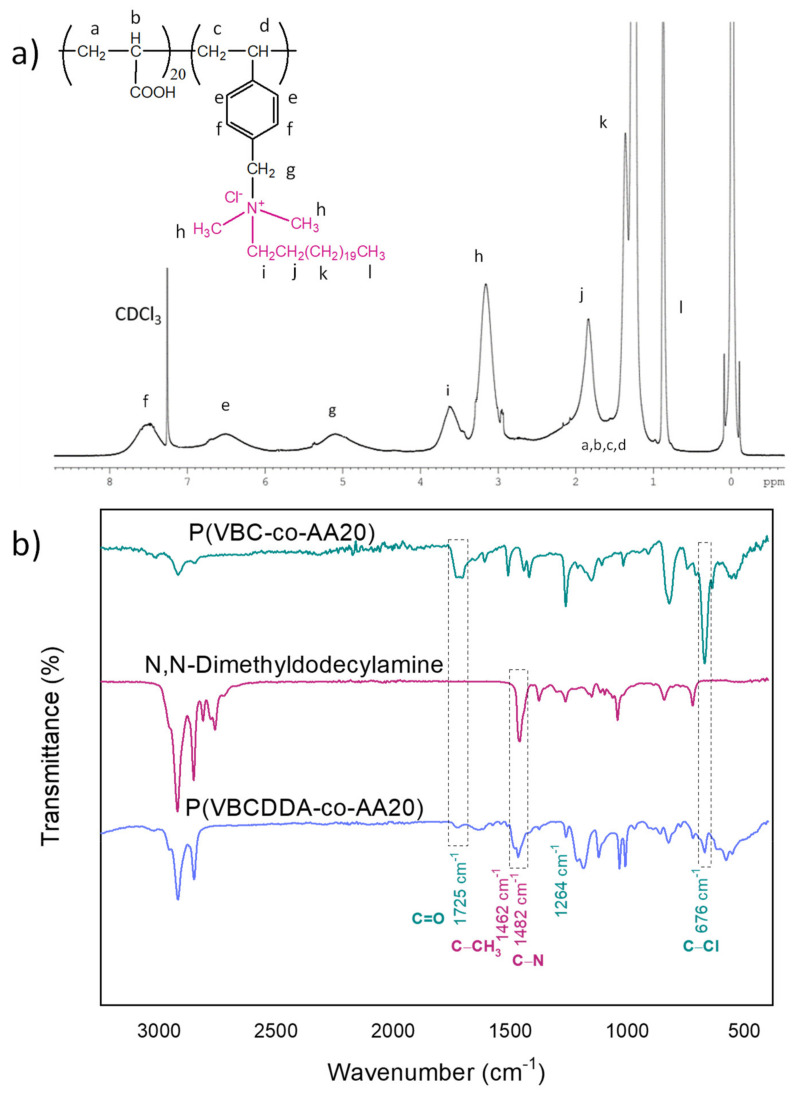
(**a**) ^1^H-NMR spectra of P(VBCDDA-co-AA20). (**b**) ATR-FTIR spectra of P(VBCDDA-co-AA20), P(VBC-co-AA20) and DDA.

For this study, the precursor copolymers P(VBC-co-AA20) and P(SSNa-co-GMA20) were synthesized through free radical polymerization as reported previously [28,31] (Scheme S1) and their chemical structures were verified through Proton Nuclear Magnetic Resonance (^1^H-NMR) and Attenuated Total Reflection Fourier Transform Infrared Spectroscopy (ATR-FTIR) spectroscopies (data not shown). Molecular weight was calculated for each copolymer through Size Exclusion Chromatography (SEC) measurements: P(VBC-co-AA20) (M_n_: 12,000, M_w_: 15,000, PDI: 1.25) and P(SSNa-co-GMA20) (M_n_: 80,000, M_w_: 150,000, PDI: 1.85). As can be observed, there is a drastic difference between the molecular weights of the two copolymers. The high values of Mw, Mn and PDI of P(SSNa-co-GMA20) are due to the Trommsdorff–Norrish effect, which induces localized increases in the viscosity of the polymerizing system leading to delayed termination reactions. This sequentially causes a rapid increase in the overall reaction rate, leading to a potential uncontrolled reaction and altering the characteristics of the produced polymer (autoacceleration) [32]. The modification of P(VBC-co-AA20) with DDA through covalent bonding was characterized by ^1^H-NMR spectroscopy on deuterated chloroform (CDCl_3_) and ATR-FTIR (Figure 1).

In the ^1^H-NMR spectrum (Figure 1a), the protons of the –CH_3_ groups associated with the nitrogen atom corresponded to the peak at 3.2 ppm, while the protons of the final –CH_3_ methyl group corresponded to the peak at 0.8 ppm. In addition, the protons of the –CH_2_– group associated with N appeared at 3.7 ppm, while the rest of the (–CH_2_–)_9_ groups were displayed at 1.3 ppm. The peaks owed to the carboxyl acid protons of AA were not obvious in the ^1^H-NMR spectrum, probably due to its lack of solubility in the organic deuterated solvent CDCl_3_.

ATR-FTIR spectroscopy for P(VBCDDA-co-AA20) (Figure 1b) showed the successful introduction of an amine, in comparison to the spectrum of precursor P(VBC-co-AA20). The observed minimization of the peaks at 676 cm^−1^ and 1264 cm^−1^ indicates the splitting of the C–Cl bond and hence the formation of a C–N bond. Moreover, two new peaks appeared at 1462 cm^−1^ and 1482 cm^−1^, which corresponded to the bending vibrations of the –CH_3_ group of the amine and the C–N bond, respectively. A strong increase of the peaks in the range of 2800–2900 cm^−1^ was also detected due to the –CH_2_– methylene groups of the long carbon chain of the amine. The carboxyl group of acrylic acid, which is the reactive part in this copolymer, can also be confirmed from the peak at 1725 cm^−1^.

**Figure 2:**
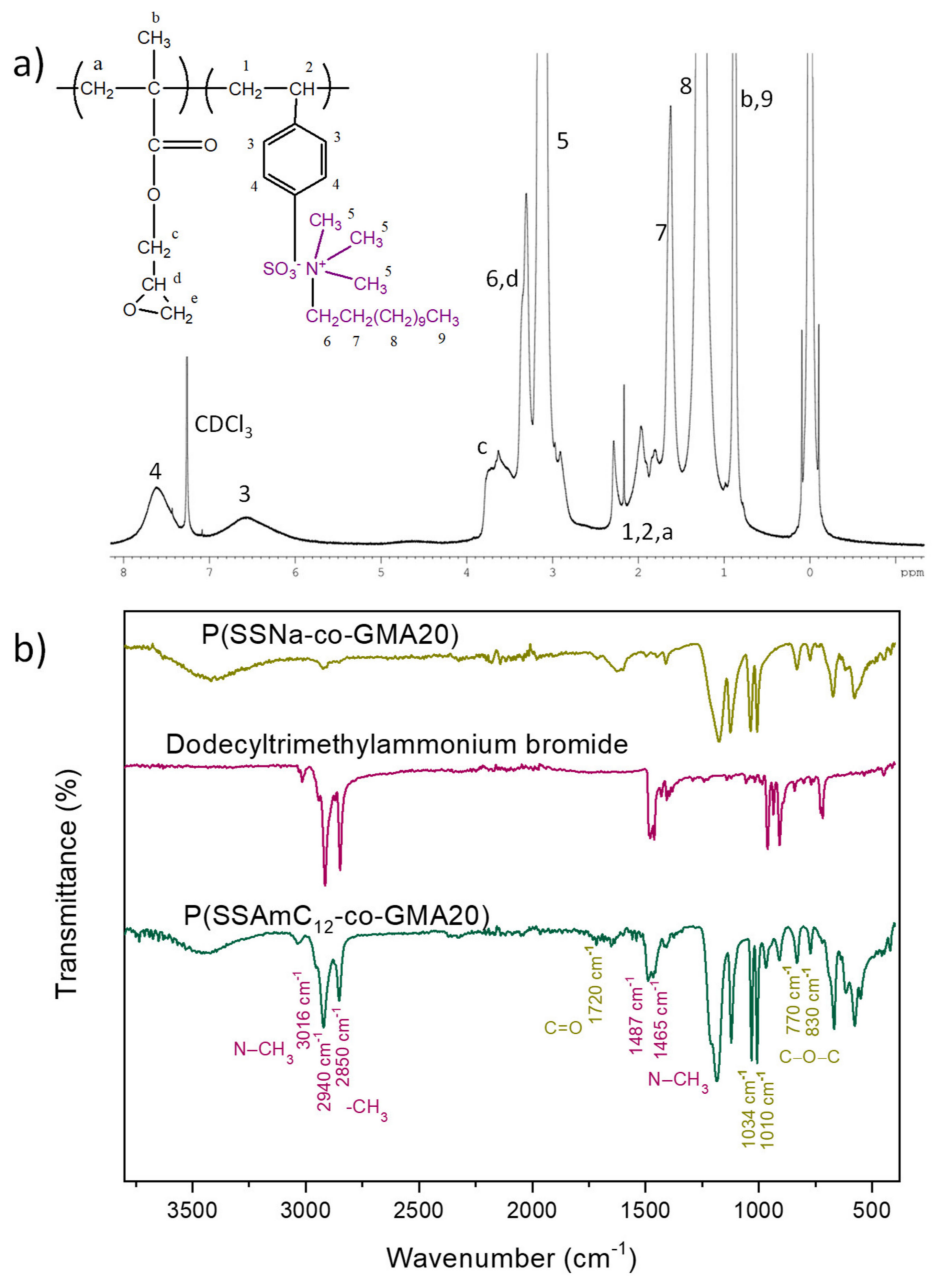
(**a**) ^1^H-NMR spectra of P(SSAmC_12_-co-GMA20); (**b**) ATR-FTIR spectra of P(SSAmC_12_-co-GMA20), P(SSNa-co-GMA20) and DTAB.

After the synthesis of P(SSNa-co-GMA20), the copolymer was modified via electrostatic attachment of AmC_12_ cations of DTAB. The final product was characterized by ^1^H-NMR spectroscopy on CDCl_3_ and ATR-FTIR (Figure 2).

More specifically, in the ^1^H-NMR spectrum (Figure 2a), the protons of the –CH_3_ groups attached to N corresponded to the peak at 3.1 ppm, while the final –CH_3_ group of the dodecyl chain appeared at 0.8 ppm. Similarly, the protons of the N-linked methylene group –CH_2_– appeared at 3.3 ppm, while the other –CH_2_– methylene groups of the aliphatic chain were displayed at 1.3 ppm. Finally, not all the proton groups of GMA were obvious in the ^1^H-NMR spectrum. Specifically, the peak at 3.6 ppm corresponded to the protons of the methylene group –CH_2_ and the peak at 2.9 ppm corresponded to the protons of the epoxy ring.

In the ATR–FTIR spectrum (Figure 2b), the characteristic peaks of the quaternary amine AmC_12_ cation and the peaks of the initial copolymer P(SSNa-co-GMA20) appeared in the spectrum of the quaternized copolymer P(SSAmC_12_-co-GMA20), confirming its successful modification. For AmC_12_ in particular, peaks at 2940 cm^−1^ and 2850 cm^−1^ were attributed to the *ν*_as_(CH_3_) and *ν*_sym_(CH_3_) modes, respectively, while the 1465 and 1487 cm^−1^ bands were characteristics of the δ_as_(N–CH_3_) mode. Moreover, the peak at 3016 cm^−1^ corresponded to the *ν*_as_(N–CH_3_) feature for the DTAB molecule, while the peaks at 1394 and 911 cm^−1^ to the δ_sym_(N–CH_3_) and *ν*(C–N) bands, respectively [33]. Finally, the peaks at the regions 830 cm^−1^ and 770 cm^−1^ corresponded to the epoxy ring, whereas the peaks at 1010 cm^−1^ and 1034 cm^−1^ were assigned to the benzene ring. The peaks at 1126 and 1182 cm^−1^ were related to the symmetric and antisymmetric vibration absorption peaks of the SO_3_ group, respectively. The peak at 1720 cm^−1^ was attributed to the carbonyl group and the peak at 676 cm^−1^ may be related to aromatic C–H out-of-plane bending (deformation) vibration [31].

The copolymers were also characterized through thermogravimetric analysis (TGA) to determine the mass reduction of the materials as a function of temperature. For the copolymer P(VBCDDA-co-AA20), the first decomposition temperature was 205 °C and the second 405 °C (Figure S1). For the copolymer P(SSAmC_12_-co-GMA20) the decomposition temperature was 340 °C.

### 2.2. Cross-Linking Reaction

**Scheme 1:**
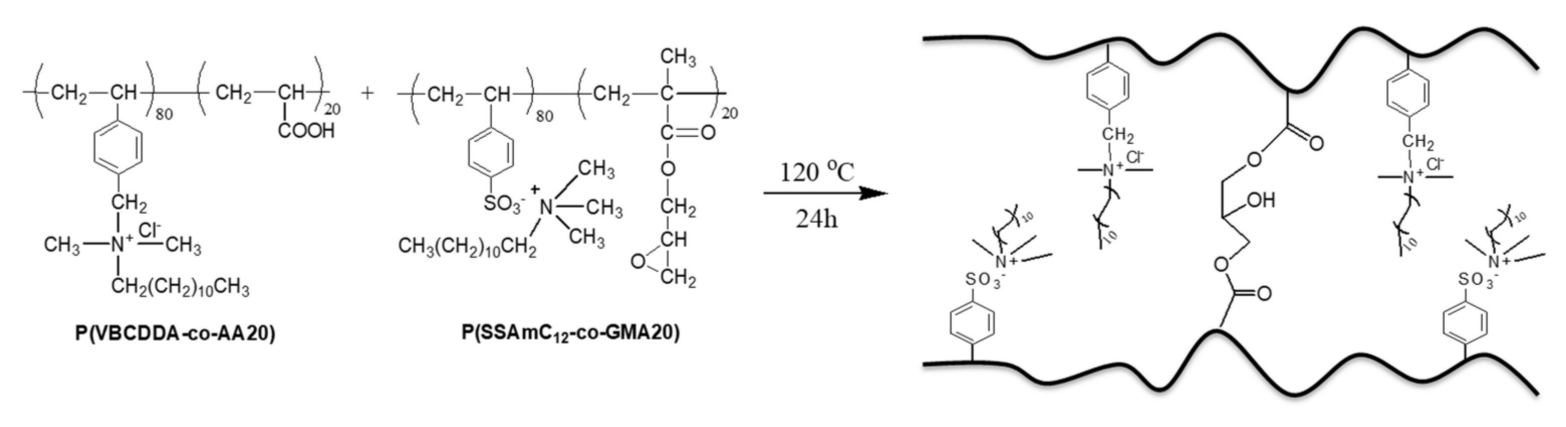
Cross-linking reaction between P(VBCDDA-co-AA20) with P(SSAmC_12_-co-GMA20).

The preparation of the polymeric mixtures P(VBCDDA-co-AA20)/P(SSAmC_12_-co-GMA20) was achieved by the reaction between the carboxyl groups of the AA unit and the epoxy groups of the GMA unit after heat treatment at 120 °C for 24 h (Scheme 1).

Different mixing ratios and solvents were tested for the preparation of the membranes. These tests and the quality of the final membranes cured at 120 °C are summarized in Table 1. The solvents used were *N*,*N*-dimethylformamide (DMF), ultrapure water (H_2_O 3D), chloroform (CHCl_3_)and ethanol (EtOH).

The systems P(VBCDDA-co-AA20)/P(SSAmC_12_-co-GMA20) 60/40 and 20/80 in CHCl_3_ showed the best miscibility and optimum membrane quality. These membranes, both as-prepared (dried at room temperature, RT) or after further treatment (cured at 120 °C) were studied in terms of their stability, by immersion in CHCl_3_, which is a good solvent for the initial copolymers. In fact, full solubility in CHCl_3_ was observed in some cases for the as-prepared membranes. In contrast, all membranes turned to partially soluble or practically insoluble after curing at 120 °C, indicating that curing was necessary to achieve a significant degree of cross-linking.

Since the intended application of the systems P(VBCDDA-co-AA20)/P(SSAmC_12_-co-GMA20) requires direct contact to water or seawater, the membranes were also immersed in pure water or aqueous 1 M NaCl solutions and left for 2 days (Table 2). Solubility tests of the copolymers indicated that the P(VBCDDA-co-AA20) remains practically insoluble in NaCl while P(SSAmC_12_-co-GMA20) remains practically insoluble in H_2_O 3D. Consequently, all membranes, especially after curing at 120 °C, appeared as partially soluble or practically insoluble in these aqueous environments.

**Figure 3:**
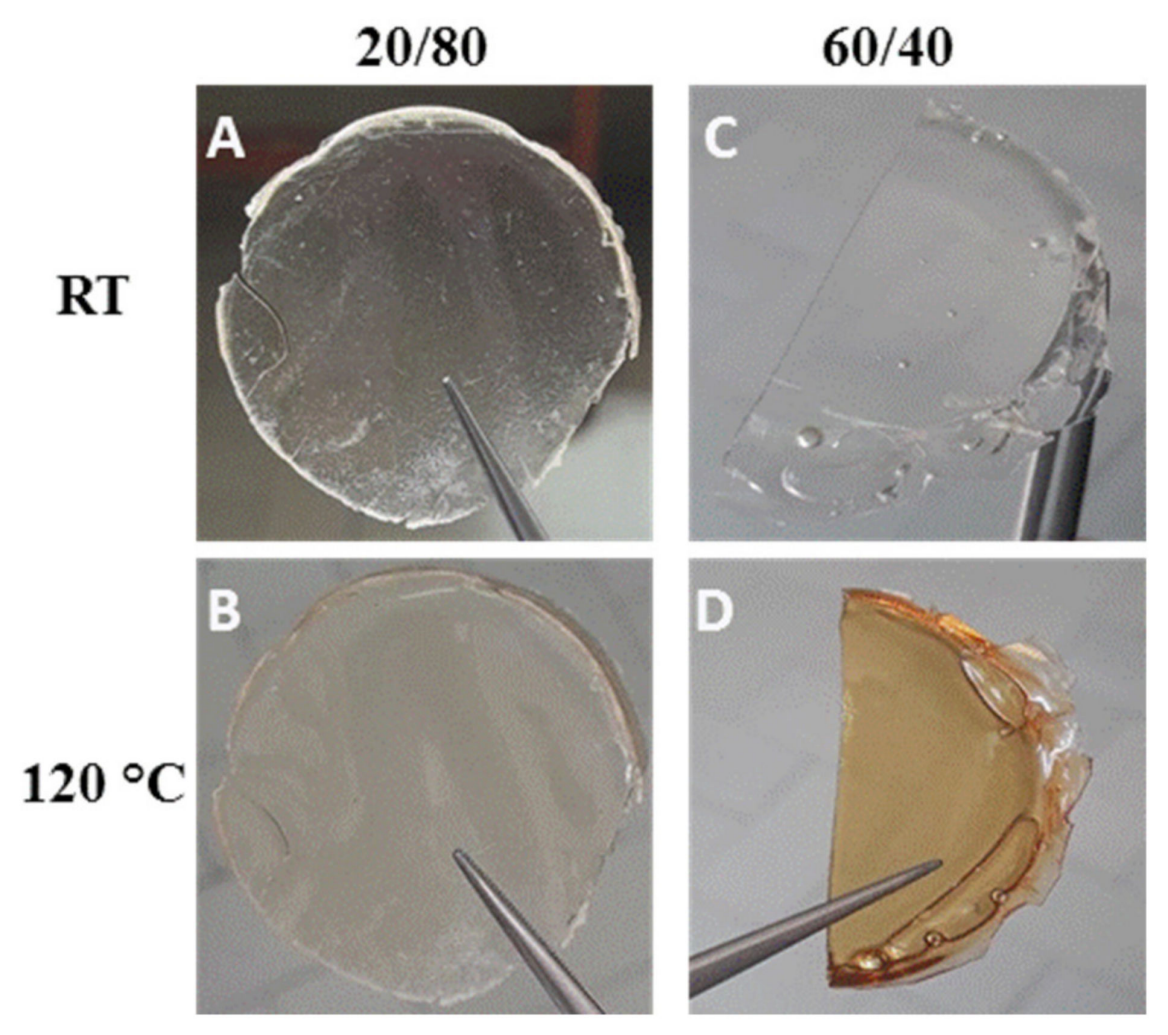
Appearance of P(VBCDDA-co-AA20)/P(SSAmC_12_-co-GMA20) 20/80 (**A**,**B**) and 60/40 (**C**,**D**) membranes, prepared at RT (**A**,**C**) and after curing at 120 °C (**B**,**D**).

Membranes P(VBCDDA-co-AA20)/P(SSAmC_12_-co-GMA20) at blend composition 20/80 and 60/40 *w*/*w* %, as-prepared (dried at RT) or cured at 120 °C, are presented in Figure 3. The membranes dried at RT appeared clear and homogeneous at both compositions, while after curing at 120 °C they acquired a yellowish color. The P(VBCDDA-co-AA20)/P(SSAmC_12_-co-GMA20) 60/40 membrane showed higher stability and flexibility.

**Figure 4:**
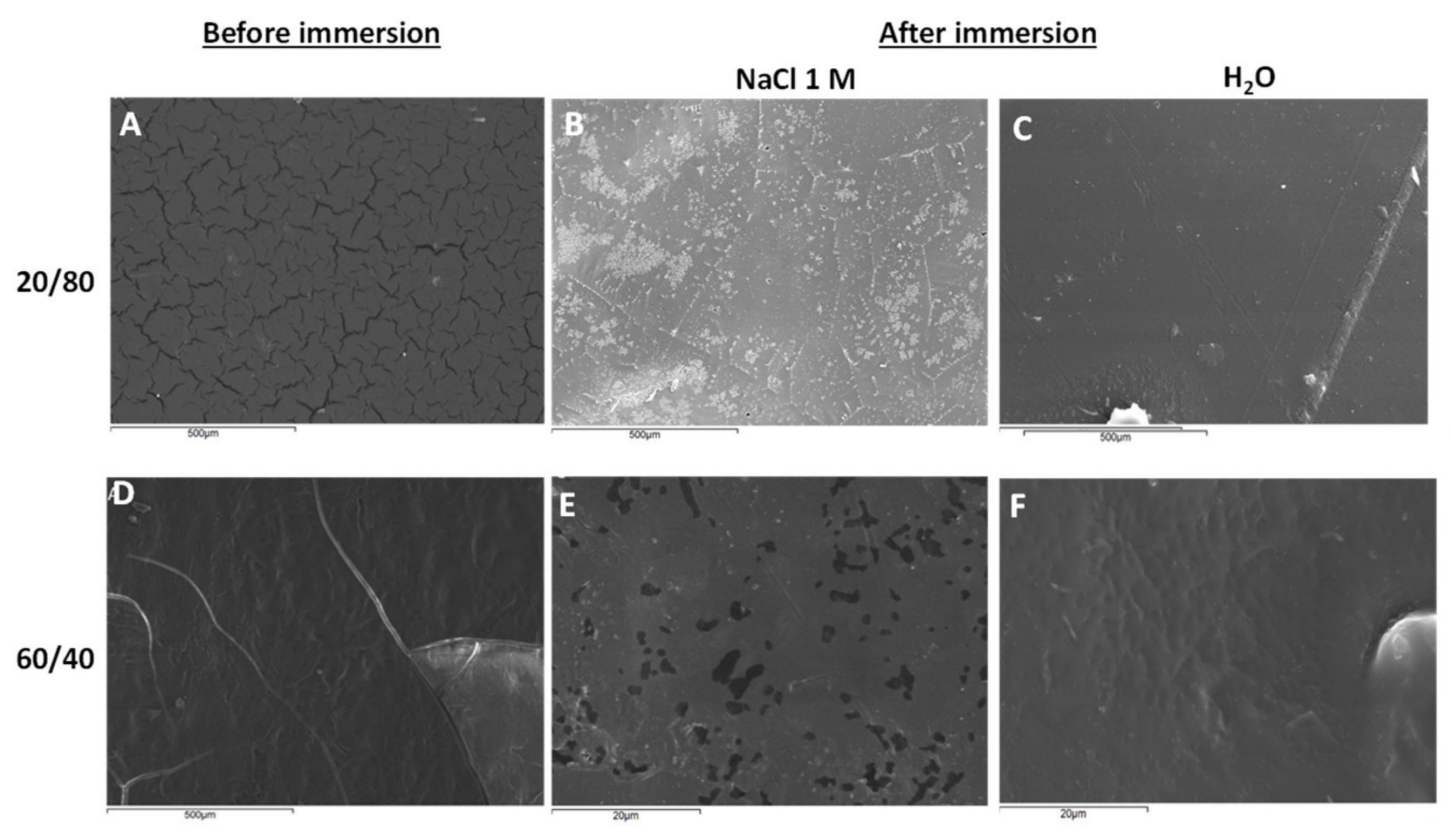
SEM images of the P(VBCDDA-co-AA20)/P(SSAmC_12_-co-GMA20) 60/40 and 20/80 membranes after 70 days of immersion in 1 M NaCl (**B**,**E**) or H_2_O 3D (**C**,**F**) and before immersion (**A**,**D**).

The membranes stood in 1 M NaCl and H_2_O 3D for 70 days. After treatment, their morphology was examined by scanning electron microscopy. The cross-linked membrane P(VBCDDA-co-AA20)/P(SSAmC_12_-co-GMA20) 60/40 was smoothed after immersion in 1 M NaCl, the effect being more pronounced in H_2_O 3D (the initial cracks of the uncross-linked membrane were not observed, Figure 4). The 20/80 membrane remained homogeneous in H_2_O 3D without cracks after 70 days, while some pores were observed in the NaCl solution indicating the release of AmC_12_ cations through ion exchange with Na^+^ cations. The membranes P(VBCDDA-co-AA20)/P(SSAmC_12_-co-GMA20) 60/40 and 20/80 were selected as the optimum systems for further studies.

### 2.3. Release Studies in Aqueous Environment

**Figure 5:**
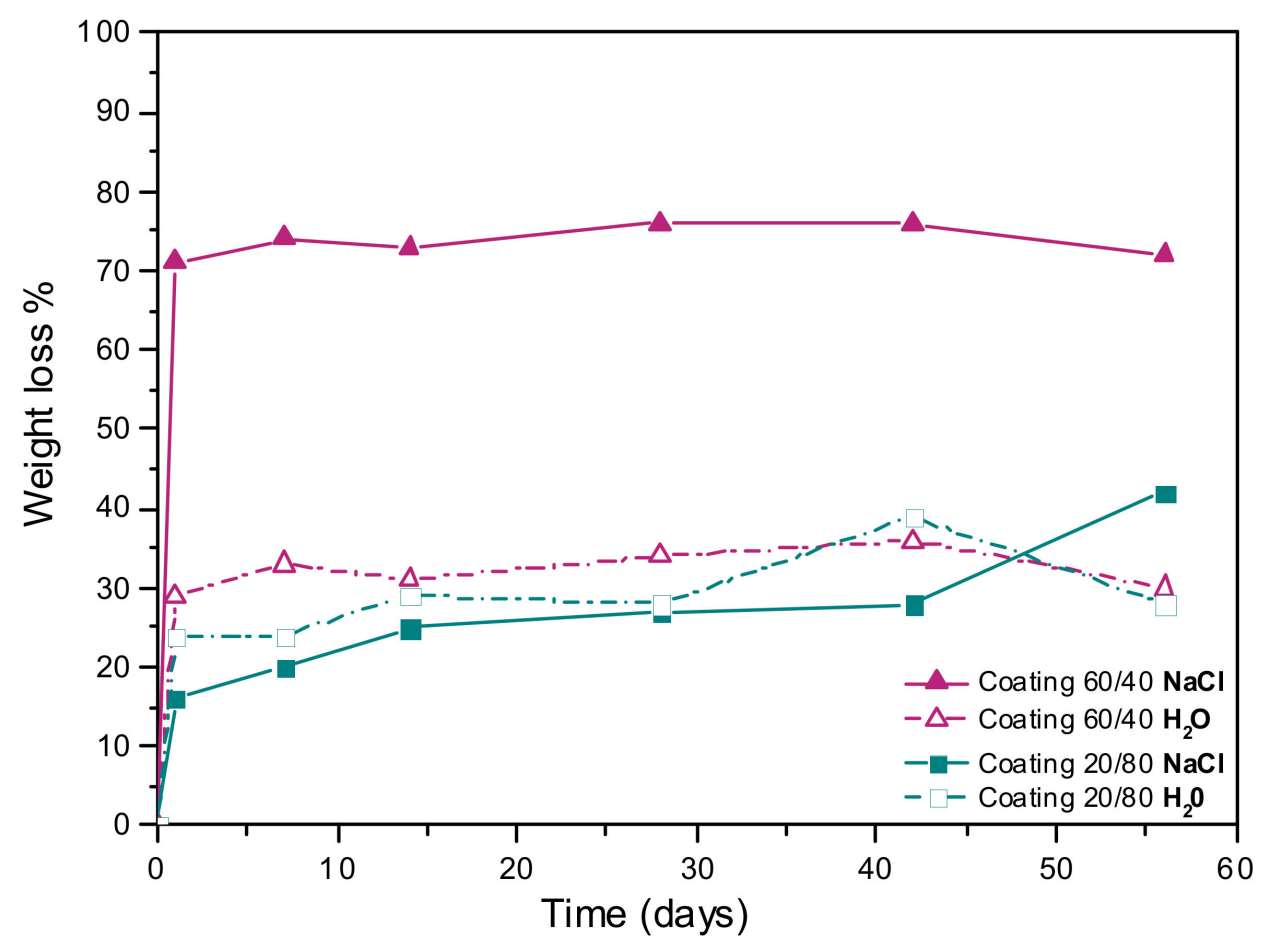
Weight loss values of cross-linked coatings P(VBCDDA-co-AA20)/P(SSAmC_12_-co-GMA20) 60/40 and 20/80 after immersion in pure water or aqueous NaCl 1 M solution, over time.

Polymeric coatings of the P(VBCDDA-co-AA20)/P(SSAmC_12_-co-GMA20) 60/40 and 20/80% ratios were prepared on glass surfaces. After cross-linking at 120 °C, the coatings were immersed in glass vials containing aqueous NaCl 1 M solutions or H_2_O 3D and left for 56 days. For the coating P(VBCDDA-co-AA20)/P(SSAmC_12_-co-GMA20) 20/80 a high weight loss was observed in the NaCl solution from the very first day, while in pure water, only 30% of the coating was dissolved (Figure 5). Apparently, a large part of the copolymer P(SSAmC_12_-co-GMA20) dissolved in salt solution, due to insufficient cross-linking. In the case of the coating P(VBCDDA-co-AA20)/P(SSAmC_12_-co-GMA20) 60/40 the release was approximately 35% from the beginning of the experiment in both H_2_O 3D and salt solution. This indicated a more successful cross-linking resulting in a more rigid and stable coating.

### 2.4. TOC and TN Measurements

**Figure 6:**
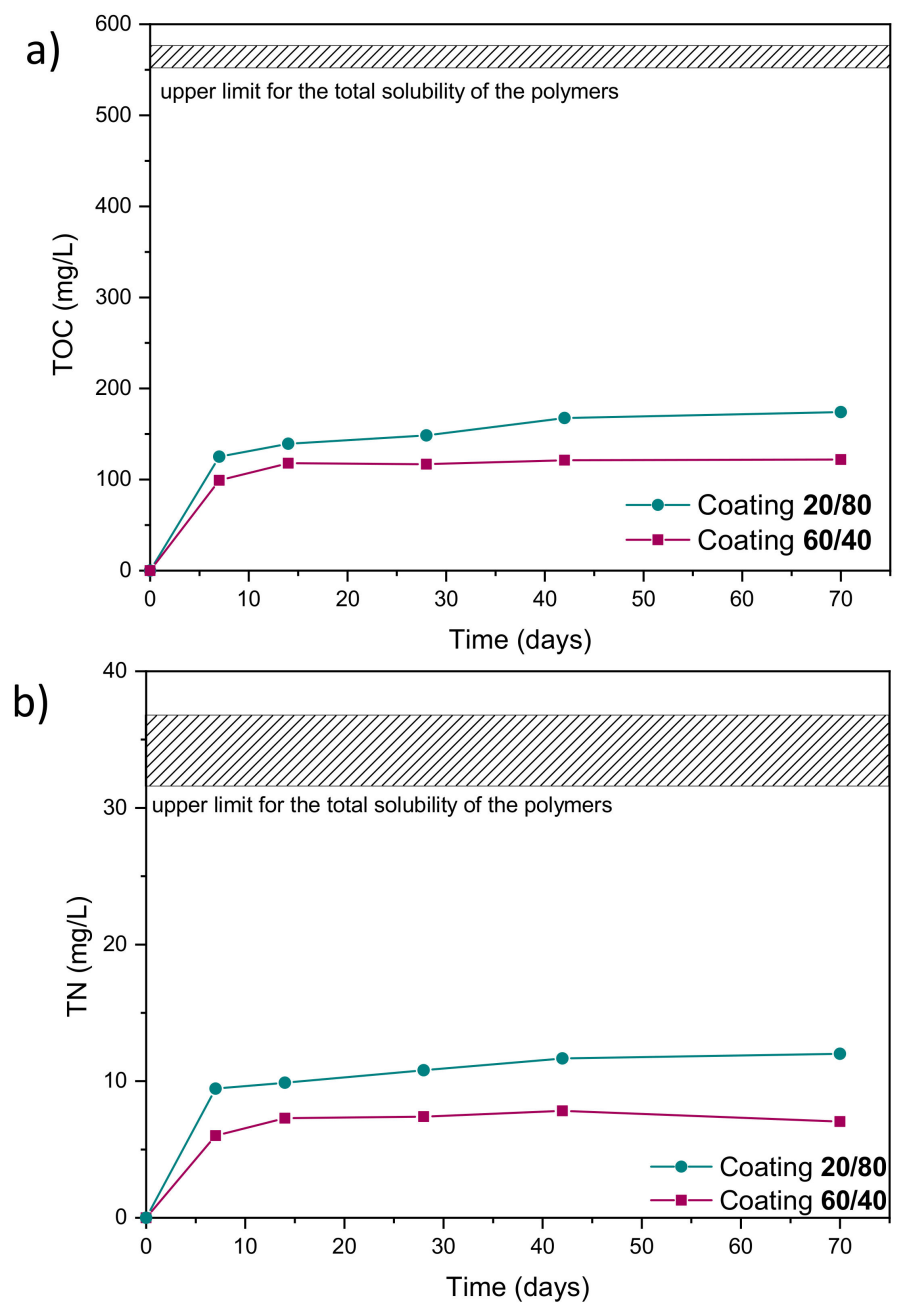
Release studies of total organic carbon (**a**) and total nitrogen (**b**) of P(VBCDDA-co-AA20)/P(SSAmC_12_-co-GMA20) coatings after immersion in pure water.

To further explore the release of these coatings in pure water, measurements of total organic carbon (TOC) and total nitrogen (TN) were made in the soluble fractions of the materials after their immersion in water (up to 70 days). Mixtures of the copolymers P(VBCDDA-co-AA20)/P(SSAmC_12_-co-GMA20) 60/40 and 20/80 *w*/*w* % ratios were examined. If the two initial copolymers P(VBCDDA-co-AA20) and P(SSAmC_12_-co-GMA20) were fully dissolved in water, the expected value of the total carbon for the 60/40 mixture would be 572 mg/L. In the case of total nitrogen the respective value would be 37 mg/L. In the case of the 20/80 mixture, the expected value for total carbon would be 553 mg/L and for total nitrogen 32 mg/L. These expected values are shown in the form of a dashed area in Figure 6. The amount of the dissolved material as calculated from the TOC/TN measurements is in a rather good agreement with the results shown in Figure 5. Specifically, in the case of the 20/80 coating, a loss of 31% was found from the TOC/TN measurements, which is in a good agreement with the respective value in Figure 5 (~30%). In the case of the coating 60/40 a loss of 20% was found from the TOC/TN measurements, a value somewhat lower than that shown in Figure 5 (30%).

**Figure 7:**
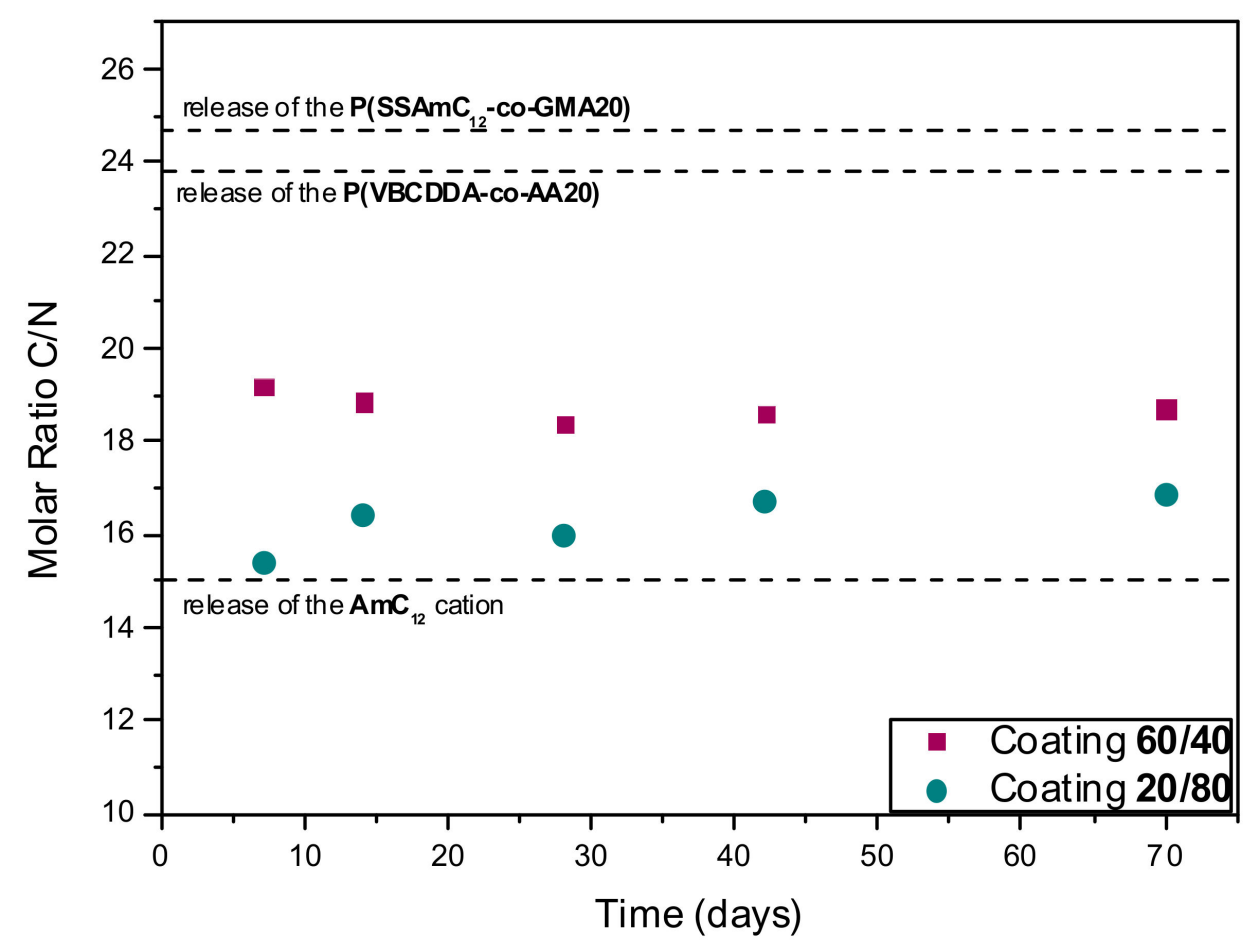
Molar ratio C/N over time, determined by TOC/TN, after immersion of blends 60/40 and 20/80 in pure water.

To determine the type of material released in the water, the C/N molar ratio was calculated for the two mixtures. The expected C/N molar ratio for the dodecyltrimethylammonium ion (AmC_12_) is 15 while for copolymers P(VBCDDA-co-AA20) and P(SSAmC_12_-co-GMA20) is 23.75 and 24.75, respectively. In the performed experiment (Figure 7), the values for the 60/40 mixture were close to 18–19 while for the 20/80 mixture they were close to 15–17. 

These values, close to the expected one for AmC_12_, suggest that the main releasable species in pure water are AmC_12_ cations. A possible explanation is an ion rearrangement, permitting the complexation of the positive VBCDDA groups and negative styrene sulfonate groups. This allows the release of AmC_12_ cations from SSAmC_12_ units with Cl^−^ anions, originating from VBCDDA units, as counterions. A similar behavior was also observed for similar systems containing hexadecyltrimethylammonium units [11]. In addition, these polymeric materials showed relatively low weight loss in pure water and much lower in a salt solution. Considering the present study, these results indicate that the decrease of the aliphatic chain length of the ammonium compound from 16- to 12-carbon atoms (and consequently the decrease of hydrophobicity) in the copolymers resulted in increased water solubility and therefore to a faster release from the cross-linked polymeric coating.

### 2.5. Antimicrobial Activity of the Polymeric Materials

**Figure 8:**
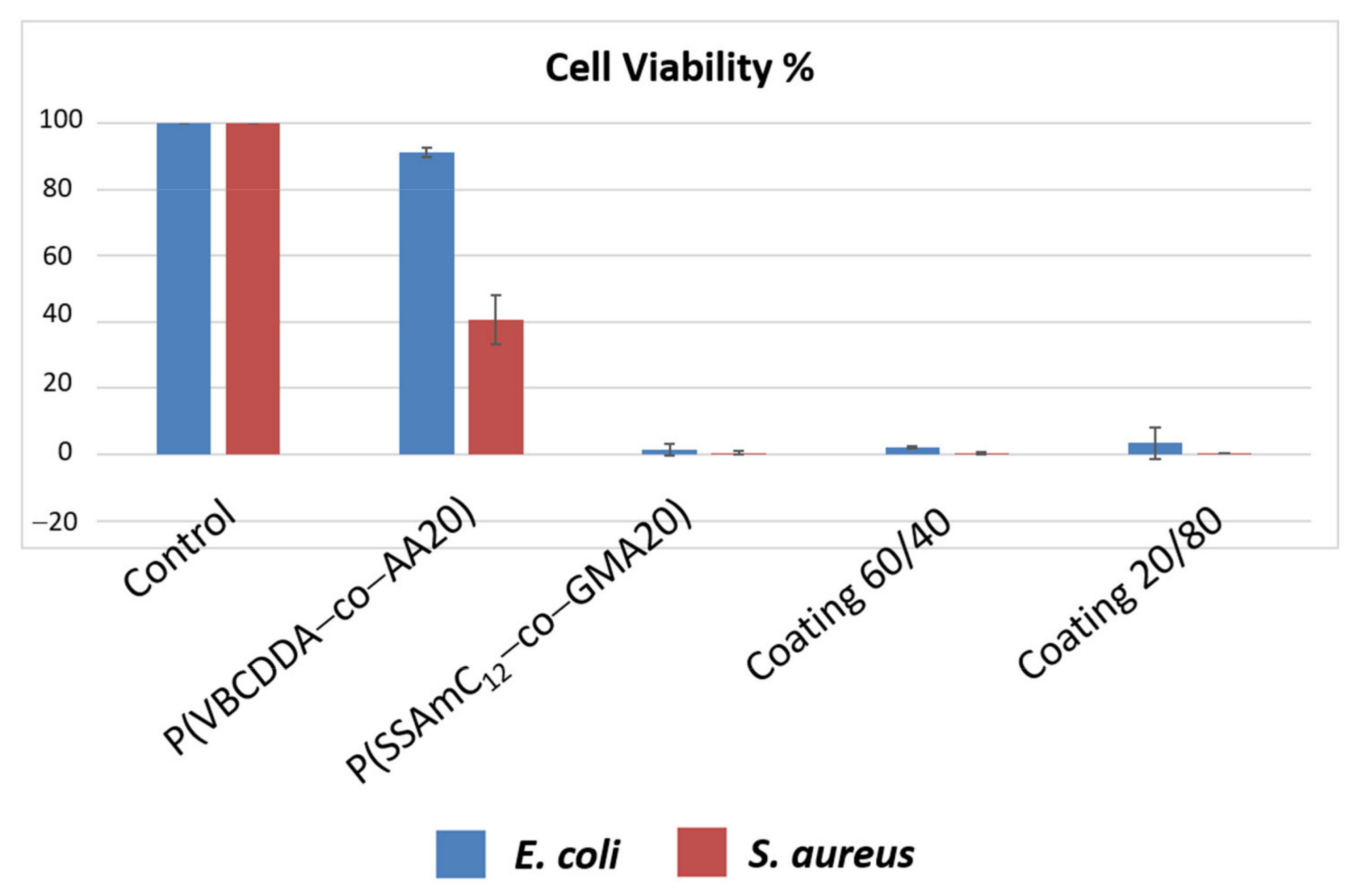
Cell viability of *E. coli* and *S. aureus* after contact with the polymeric materials. The Y axis demonstrates cell viability (%).

The antimicrobial activity of the quaternary dodecyl-ammonium-modified copolymers and their blends was evaluated against *E. coli* and *S. aureus* (Figure 8).

The initial copolymers P(VBCDDA-co-AA20) and P(SSAmC_12_-co-GMA20) exhibited a very different activity against *E. coli* and *S. aureus* after 2 h of contact. Specifically, the P(SSAmC_12_-co-GMA20) copolymer was almost equally active against both bacteria (98.7% reduction of *E. coli* cells and 99.5% reduction of *S. aureus* cells), whereas the P(VBCDDA-co-AA20) copolymer was ineffective against *E. coli* (8.9% cell reduction) but more effective against *S. aureus* (59.3% cell reduction). This difference is most likely attributed to how the polymers may have affected the outer surfaces of Gram-positive and Gram-negative bacteria [34]. In the Gram-positive bacteria, the cytoplasmic membrane is surrounded by a rigid peptidoglycan layer with plenty of pores allowing for external molecules to encounter the cytoplasmic membrane. In the Gram-negative bacteria, the peptidoglycan layer is not in contact with the solvent but is surrounded by a second outer membrane that may act as an extra barrier hindering the diffusion of foreign molecules, especially those with a high molecular weight. The additional outer membrane of the Gram-negative bacteria may therefore explain why P(VBCDDA-co-AA20) was inactive against *E. coli*, in contrast to P(SSAmC_12_-co-GMA20), which works through the release of the small molecular weight cationic biocide DTAB. In any case, the combination of the two copolymers in compositions 60/40 and 20/80 *w*/*w* % maintained their biocidal activity against both bacteria (Figure 8). The 60/40 *w*/*w* % ratios led to a 97.8 and 99.7% mortality for *E. coli* and *S. aureus*, respectively, whereas the coating 20/80 *w*/*w* % resulted in a 96.6 and 99.8% cell reduction.

The main purpose of this work was to better understand how the hydrophilicity/hydrophobicity ratio of an antimicrobial compound may affect its effect. It is well known that the antimicrobial efficacy of cationic polymers can be improved by increasing their hydrophobicity [35]. The antimicrobial results presented here are compared to previous published work, where similar copolymers bearing more hydrophobic aliphatic chains with 16-carbon atoms were studied [27,28]. More specifically, in the case of the SSAmC_16_-based polymers, the killing efficacy was significantly higher against both *E. coli* and *S. aureus* after 3 h of contact. This result was expected due to the biocidal group hexadecyltrimethylammonium bromide (CTAB), which was electrostatically attached to the polymeric backbone, and in aqueous conditions can be released and diffused easily through the bacterial cell wall. On the other hand, the copolymer P(VBCHAM-co-AA12), where the quaternary vinylbenzyl hexadecyldimethylammonium unit bears the 16-carbon chain covalently bound, exhibited a negligible activity against *E. coli* and a slight activity against *S. aureus*. Finally, the cross-linked polymeric membranes P(SSAmC_16_-co-GMA6)/P(VBCHAM-co-AA5.5) of composition 40/60 and 20/80 *w*/*w* % presented very high antimicrobial activity against both Gram-negative and Gram-positive bacteria. The bactericidal activities observed for the polymeric materials of this work denote that the antimicrobial activity is not affected significantly by decreasing the carbon atoms of the alkyl chains from 16 to 12.

## 3. Materials and Methods

### 3.1. Materials

The monomers sodium 4-styrenesulfonate (SSNa), glycidyl methacrylate (GMA), acrylic acid (AA) and 4-vinylbenzyl chloride (VBC), the amine *N*,*N*-dimethyldodecylamine (DDA), the initiator azodiisobutyronitrile (AIBN), the deuterated chloroform (CDCl_3_) and deuterium oxide (D_2_O), as well as the solvents *N*,*N*-dimethylformamide (DMF) and hexane were purchased from Aldrich (Aldrich, Steinheim, Germany) and used as received. The solvents dimethyl sulfoxide (DMSO), chloroform (CHCl_3_), ethanol (EtOH), methanol and ethyl acetate were supplied by Fischer Scientific (Fisher Scientific, Pittsburgh, PA, USA). Acetone was purchased from Chem-Lab (CHEM-LAB NV, Zedelgem, Belgium). Quaternary surfactant dodecyltrimethylammonium bromide (DTAB) was a product of Acros Organics (Thermo Fisher Scientific, Merelbeke, Belgium). Ultrapure water (H_2_O 3D) was prepared by the SG water purifier.

### 3.2. Synthesis of Copolymers

The copolymers poly(4-vinylbenzyl chloride-co-acrylic acid) and poly(sodium 4-styrenesulfonate-co-glycidyl methacrylate) were synthesized through free radical copolymerization using AIBN as initiator. The experimental procedures have been previously described [31,36]. Briefly, the desired quantities of the two monomers (total monomer concentration 1 M) were dissolved in CHCl_3_ and DMSO, respectively, the solutions were degassed, and the initiator AIBN (0.8 mol % over the total monomer concentration) was added. The reaction was left to proceed overnight under vigorous stirring in Ar atmosphere in an oil bath set at 60 or 80 °C, respectively. The copolymers were recovered by precipitation in ethyl acetate and acetone, respectively, filtered and dried in a vacuum oven at 60 °C for 24 h. Copolymers with AA and GMA molar content of 20% were prepared. The content of AA and GMA was determined from ^1^H-NMR spectroscopy using CDCl_3_ or D_2_O as solvent, respectively. The copolymers are denoted as P(VBC-co-AA20) and P(SSNa-co-GMA20), respectively.

### 3.3. Post Polymerization Quaternization

#### 3.3.1. Synthesis of P(VBCDDA-co-AA20)

In a 100 mL round bottom flask, 2 g of P(VBC-co-AA20) was dissolved in 20 mL of CHCl_3_. Then, 3.49 mL of DDA was added. The flask was then capped, and the reaction mixture was stirred at 75 °C for four days. Precipitation in 200 mL of hexane followed. The final product was obtained by filtration and dried under vacuum for 24 h at 60 °C. Characterization of the quaternized copolymer was performed by ^1^H-NMR spectroscopy in CDCl_3_ and ATR-FTIR spectroscopy.

#### 3.3.2. Synthesis of P(SSAmC_12_-co-GMA20)

A quantity of 5 g P(SSNa-co-GMA20) and 125 mL H_2_O 3D were placed in a 500 mL round bottom flask and left under stirring at 50 °C until complete dissolution of the copolymer. In a 250 mL conical flask, 7.69 g of DTAB was dissolved in 77 mL of H_2_O 3D at 50 °C. Then, the DTAB solution was introduced dropwise into the flask that contained the copolymer and the solution was stirred at room temperature for 24 h. The precipitated final product was recovered through filtration and dried under vacuum at 60 °C for 24 h. The copolymer was characterized by ^1^H-NMR spectroscopy in CDCl_3_ and ATR-FTIR spectroscopy.

### 3.4. Cross-Linking Reaction of P(VBCDDA-co-AA20)/P(SSAmC_12_-co-GMA20)

To test the cross-linking reaction between carboxyl and epoxy groups, membranes of the two quaternized copolymers were prepared through solution casting. For this purpose, the copolymers P(VBCDDA-co-AA20) and P(SSAmC_12_-co-GMA20) were initially dissolved in organic solvent CHCl_3_, at a concentration of 5 *w/v* %. The solutions were mixed at the desired mixing ratio and the final blends were left under stirring until complete homogenization. Afterwards, the blends were poured onto glass petri dishes and left at room temperature for 24 h for solvent evaporation. The next day, the formed membranes were placed in an oven at 120 °C and remained for 24 h for cross-linking. Various blend compositions (*w*/*w* %) of the two copolymers were tested to find the optimum cross-linking conditions. The tested parameters are listed in Table 1.

### 3.5. Preparation of Samples for Solubility and Release Studies

Small pre-weighed pieces of the cross-linked membranes were placed in glass vials containing CHCl_3_ (the solvent used for membrane preparation), and left at room temperature for 48 h. The membranes were removed from the solvent, dried and weighed in order to check weight losses. Moreover, cross-linked coatings on glass surfaces were immersed in pure water or aqueous 1 M NaCl solutions and left for 3 months. Coatings were then removed from the solutions, washed (in the case of NaCl solution) and dried. The soluble fractions of the two membranes in pure water or aqueous 1 M NaCl solution were then evaluated through gravimetry and TOC/TN measurements in the final solutions.

### 3.6. Chemical Characterization

#### 3.6.1. Proton Nuclear Magnetic Resonance (^1^H-NMR)

The synthesized polymers were characterized by ^1^H-NMR spectroscopy using the Bruker AVANCE DPX 400 MHz spectrometer. CDCl_3_ and D_2_Owere used as solvents.

#### 3.6.2. Attenuated Total Reflection Fourier Transform Infrared Spectroscopy (ATR-FTIR)

The ATR-FTIR spectra of the copolymers were recorded using a Bruker Platinum ATR-FTIR spectrometer.

#### 3.6.3. Size Exclusion Chromatography (SEC)

For organically soluble polymers, SEC measurements were performed at 25 °C and flow rate of 1 mL/min with CHCl_3_ as eluent. A Polymer Lab chromatograph equipped with Ultra Styragel columns (104, 500 Å), a UV detector (254 nm) and a refractive index detector were used. Standard polystyrene (PS) samples were used to calibrate the columns. A Millipore Waters 501 HPLC chromatograph was used for the water-soluble polymers, equipped with two Shodex B-804 and B-805 columns (8 mm × 500 mm) and a differential refractive index detector (R401). Standard (ethylene oxide) samples were used to calibrate the columns. Measurements were performed at 25 °C with 0.1 M LiNO_3_ as eluent and a flow rate of 1 mL/min.

#### 3.6.4. Total Organic Carbon (TOC) and Total Nitrogen (TN) Measurements

The analyses of TOC and TN were conducted using a Schimadzu TOC analyser (TOC-VCSH) coupled to a chemiluminescence detector (TNM-1 TN unit) [37].

#### 3.6.5. Scanning Electron Microscopy (SEM)

The Zeiss SUPRA 35VP model, equipped with an EDS detector, was used to study membrane surfaces.

#### 3.6.6. Thermogravimetric Analysis (TGA)

TGA analysis was conducted in the temperature range from room temperature to 800 °C under nitrogen atmosphere and a heating rate 20 °C min^−1^ using a Labsys TG instrument (Setaram Instrumentation, Caluire, France).

### 3.7. Antimicrobial Activity Test

#### 3.7.1. Bacterial Culture Preparation

*Escherichia coli* (*E. coli*, MC1061 strain, lab collection) and *Staphylococcus aureus* (*S. aureus*, NCTC 6571 strain, (Health Protection Agency, Porton Down, Salisbury, UK) were used as representatives of Gram-negative and Gram-positive test-organisms to test the antimicrobial activity of the two copolymers and their blends. Cultures to provide bacteria for viability assays were set in LB (8 mL) from single colonies and left for approximately 18 h (overnight) at 37 °C in 15 mL tubes placed horizontally, at 80 rpm (final cell density of approximately 10^8^–10^9^ cfu·mL^−1^).

#### 3.7.2. Cell Viability on Polymeric Coatings

In a first step, glass coupons (18 × 18 mm) were coated with the polymeric solutions under sterile conditions and left to dry at room temperature, RT, overnight. Next, 20 μL aliquots of overnight cultures of each bacterial species were placed on each of the glass coupons coated with the polymers for 2 h at 22 °C, after which each coupon was transferred into a sterile 50 mL tube containing 30 mL of LB broth. The tubes were incubated at 80 rpm placed horizontally, at 37 °C for 8 h for *E. coli* and 24 h for *S. aureus*. Cell growth (scattering) was measured at 600 nm, diluting if needed in water so that the final A_600_ remained ≤0.5. The volume of inoculation (20 μL) and the time of growth for each species were optimized to ensure that all growth measurements were performed when the *E. coli* and *S. aureus* controls (no polymer exposure) were in their exponential phase of growth (Figure S2). Each experiment was replicated at different days with bacteria from different starting cultures. The effect of the polymers on cell viability was estimated by comparing the growth of the controls to that of cells exposed to polymers by the following equation:

(1) $$\mathrm{Cell}\;\mathrm{Viability}\%=\frac{{\mathrm{OD}}_{600}\;\mathrm{Sample}}{{\mathrm{OD}}_{600}\;\mathrm{Control}}\times 100\%$$

## 4. Conclusions

Firm polymeric membranes and coatings based on covalently and electrostatically bound dodecyl-ammonium groups at various composition ratios were developed and cross-linked through ring-opening of the epoxide unit of GMA (P(SSAmC_12_-co-GMA20)) by the carboxylic acid of AA (P(VBCDDA-co-AA20)) after heat treatment.

The main goal of this study was to examine how hydrophobicity might affect the antibacterial and solubility properties of the novel polymers. In terms of stability, the decrease of the aliphatic chain of the ammonium compound from 16- to 12-carbon atoms, increased the water solubility of the original copolymers and therefore their faster release from the cross-linked polymeric coating. Regarding their antimicrobial efficacy against *E. coli* and *S. aureus*, the decrease of hydrophobicity did not affect the high levels of biocidal activity of the complementary copolymers, nor those of the final cross-linked materials.

We believe that such polymers, exhibiting strong antimicrobial activity and good solubility in aqueous environment, are promising candidates for antimicrobial and antifouling applications. The novel coatings combine contact- and release-based antimicrobial activity.

## Figures and Tables

**Table 1:** Examined parameters for the preparation of P(VBCDDA-co-AA20)/P(SSAmC_12_-co-GMA20) membranes at concentration 5 *w*/*v* % and curing temperature of 120 °C.

Membranes’ Code	Equivalents’ Ratio (eq AA/eq GMA)	Solvent	Composition (*w*/*w* %)	Quality ^a^
M1	1/1	EtOH	60/40	+
M2	1/1	H_2_O 3D	60/40	+
M3	1/1	DMF	60/40	+
M4	1/1	CHCl_3_	60/40	+++
M5	4/1	CHCl_3_	80/20	++
M6	1/4	CHCl_3_	20/80	+++

^a^: +++, very good; ++, good; +, mediocre.

**Table 2:** Membrane solubility of 1 *w/v* % membrane material in CHCl_3_, aqueous NaCl 1 M solution and H_2_O.

Code	Dried at RT			Cured at 120 °C		
	CHCl_3_	NaCl 1 M	H_2_O 3D	CHCl_3_	NaCl 1 M	H_2_O 3D
M4	Partially soluble	Partially soluble	Partially soluble	Insoluble	Insoluble	Insoluble
M5	Soluble	Partially soluble	Soluble	Partially soluble	Partially soluble	Partially soluble
M6	Partially soluble	Partially Soluble	Partially soluble	Partially soluble	Partially soluble	Insoluble

## Data Availability

Not applicable.

## Supplementary Materials

The following are available online at https://www.mdpi.com/article/10.3390/ijms222413236/s1.
