# Supplementary material for: Preparation of Antimicrobial Coatings from Cross-Linked Copolymers Containing Quaternary Dodecyl-Ammonium Compounds
Source: Int J Mol Sci. 2021 Dec 8;22(24):13236. doi: 10.3390/ijms222413236 (PMC8707885; doi:10.3390/ijms222413236)
Supplement: Supplementary file 1 [file ijms-22-13236-s001.zip › ijms-1489355-supplementary.pdf]

International Journal of
Molecular Sciences

Supplementary Materials

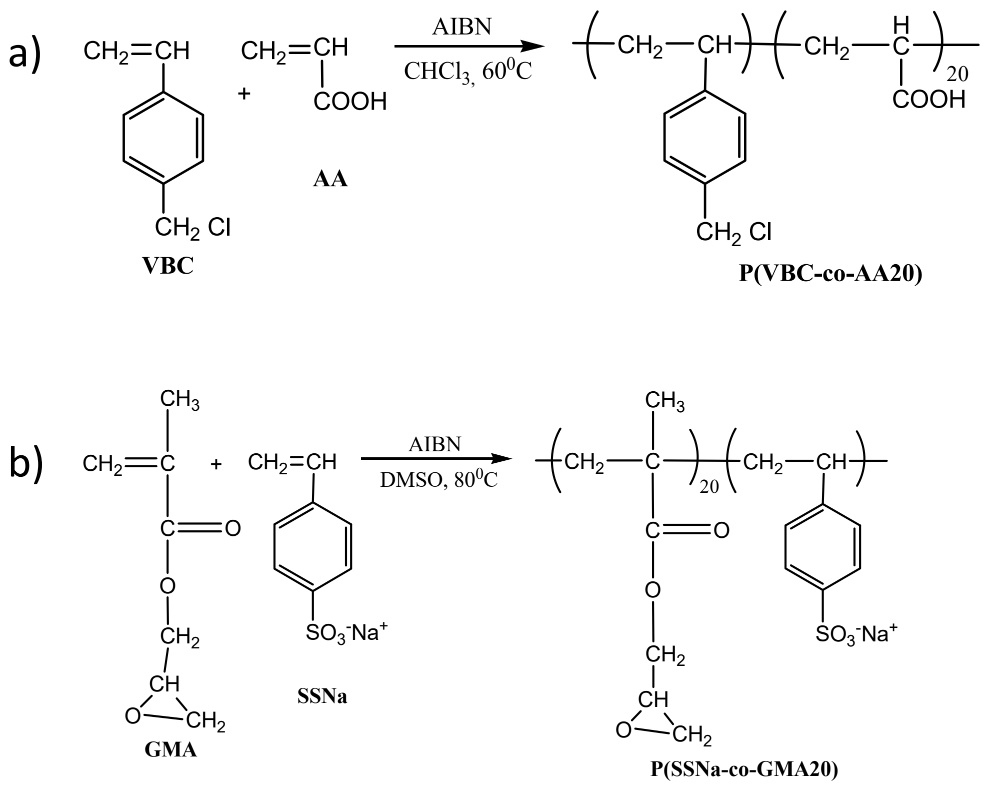

Scheme S1. Polymerization reaction of P(VBC-co-AA20) (**a**) and P(SSNa-co-GMA20) (**b**).

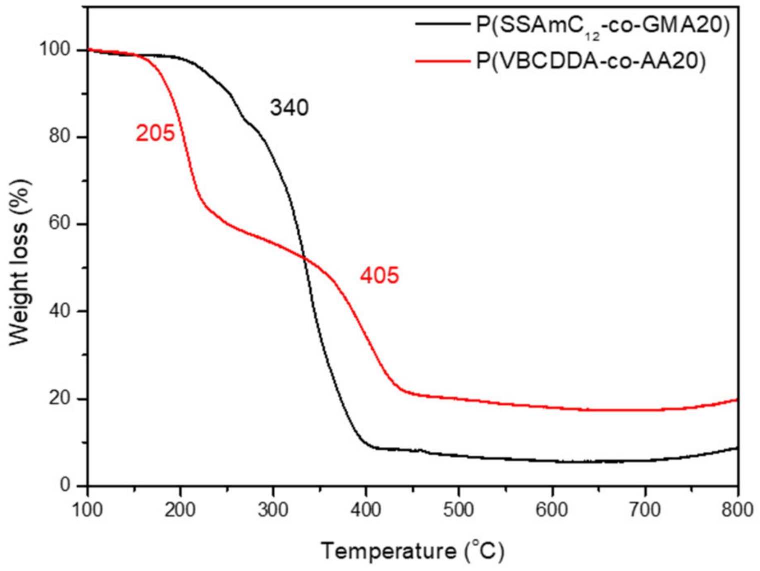

Figure S1. Thermogravimetric analysis of P(VBCDDA-co-AA20) and P(SSAmC12-co-GMA20).

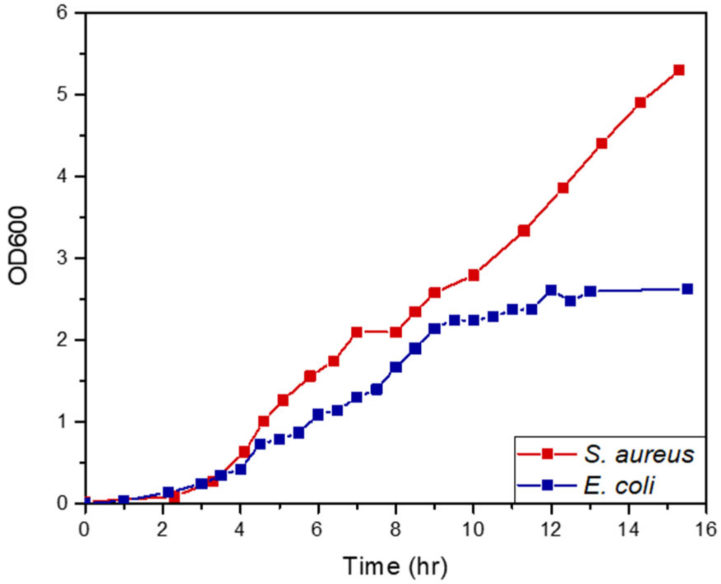

Figure S2. Growth curves of *S. aureus* (red) and *E. coli* (blue) in LB medium. Cells were grown in 30 mL LB medium at 80 rpm in 50 mL sterile tubes placed horizontally at 37 °C. For the *E. coli* and *S. aureus* cultures, inoculi were 100 and 200 μL respectively from overnight cultures at 37 °C.
